# Storytelling and training to advance individual recovery skills (STAIRS). A feasibility study of a blended program to support personal recovery among patients with a major depressive disorder in remission

**DOI:** 10.3389/fpsyt.2022.984104

**Published:** 2022-09-23

**Authors:** David Wedema, Joanieke Siero, Eliza L. Korevaar, Klaas J. Wardenaar, Manna A. Alma, Robert A. Schoevers

**Affiliations:** ^1^Research and Innovation Center for Rehabilitation, Hanze University of Applied Sciences, Groningen, Netherlands; ^2^Research School of Behavioural and Cognitive Neurosciences (BCN), Department of Psychiatry, University of Groningen, University Medical Center Groningen, Groningen, Netherlands; ^3^Department of Health Sciences, Applied Health Research, University of Groningen, University Medical Center Groningen, Groningen, Netherlands

**Keywords:** depression, rehabilitation, peer group, social support, psychosocial functioning, empowerment

## Abstract

Because major depressive disorder (MDD) has a strong negative impact on patients' lives, well–designed treatment programs are needed that address the lasting effects of MDD. Previous work has shown that such programs should not only focus on symptomatic recovery, but also on the subsequent personal recovery process. Currently, few programs with this specific focus exist. Therefore, this study aimed to assess the feasibility of a newly developed blended program to support the personal recovery process of MDD patients: Storytelling and Training to Advance Individual Recovery Skills (STAIRS). STAIRS is a program using peer support and guidance by experts by experience and clinicians, which can be added to regular depression treatment when symptomatic recovery is almost reached. Topics addressed in this program are: (1) effects of depression and treatment; (2) structure; (3) (self) stigma; (4) self-image; (5) meaning of life; (6) connection to others; (7) physical health; (8) relaxation; and (9) preventing relapse. Experiences with the STAIRS program were collected from five participating patients with questionnaires and a focus-group interview, as well as from four trainers using semi-structured interviews. Participants valued the topics addressed in STAIRS, the used working methods, the presence of an expert by experience and the ability to share experiences with peers. The use of an online platform and the involvement of others is seen as potentially supportive but turned out to be more challenging. Perceived effects of STAIRS include positive changes in participants' daily lives and their contacts with others. Overall, the results indicate that when implemented accessibly, STAIRS is a promising and feasible program to foster personal recovery among patients recovering from MDD.

## Introduction

Major depressive disorder (MDD) is one of the major contributors to the global burden of disease (1), affecting approximately 280 million people in the world (2). One in five persons experience at least one depressive episode in their lifetime (3), after which it is highly recurrent. After the first episode, there is a 60% lifetime risk of recurrence, which increases to 70% after two episodes and 90% after three or more previous episodes (4). One factor contributing to this is that almost half of all patients experience residual symptoms during periods of remission (5), which is a known predictor of subsequent relapse and recurrence (6). Residual symptoms can lead to long-term psychosocial impairments (7), which in turn increase the chance of relapse (8). This means that sufficient attention should be paid to the question of how to cope with the lasting effects of MDD when patients enter the last phase of depression treatment.

Recent standardized guidelines for the treatment of MDD have adopted a broader view on recovery, which goes beyond merely the recovery from symptoms alone (9–11). Equal importance is given to *personal recovery*, which is defined as the unique process of finding a new balance in life wherein someone is satisfied, hopeful and feels they have a meaningful life, even when there are limitations caused by illness (12). Previous research has shown that personal recovery is weakly to moderately associated with symptomatic recovery (13–15), but continues beyond the illness as the process of finding new ways to live with (residual) symptoms (16). One important aspect in this process is the impact of the illness on one's identity, which affects their hope and self-esteem and can lead to lasting difficulties in coping with daily tasks, social interactions and vocational outcomes (17). As a result, personal recovery can lag behind symptomatic recovery by up to several years (18–20). A focus on personal recovery after symptomatic recovery is thus needed during treatment to address more completely the negative impact of MDD on patients' lives (21).

To support personal recovery, Leamy et al. (22) elegantly combined the underlying elements in the comprehensive CHIME framework, which states that to recover from a mental illness patients need (1) *Connectedness* with others, (2) *Hope* that there are possibilities to get better, (3) a redefined *Identity* wherein there is balance between vulnerabilities and possibilities, (4) *Meaning* to life (including the ability to fulfill significant roles in life), and (5) a sense of *Empowerment*. Two factors can be distinguished that support these elements. First, the involvement of others is an important factor (9, 23, 24). Family support can contribute to hope, encouragement, seeing opportunities and getting into community activities (25). In addition, peers can provide insight into possible ways to cope with difficulties, encourage patients to actively try new strategies and enhance their problem-solving skills (26, 27). Peers with lived experience (i.e., “experts by experience”) can also play an important role by using their own experiences to support other patients in their recovery process and by providing hope (28). The second factor is adding the use of online facilities to face-to-face contact (blended care). This may strengthen patients' empowerment and facilitate the involvement of significant others (29). The ability to continue working on goals independently online increases patients' self-management (30) and helps to maintain what has been achieved in face-to-face contacts (31).

However, despite the emergence of a broader view on recovery, most depression treatments still focus mainly on symptomatic recovery (32). This is also apparent from the observed gap between the concept of personal recovery on the one hand and the actual routine provision of personal recovery orientated interventions on the other hand (23). In addition, available interventions that do focus on personal recovery in psychiatry are mostly intended for patients suffering from schizophrenia or other persisting severe mental illnesses (14). Some recovery orientated interventions for patients with MDD have been developed and well-studied. These include the Wellness Recovery Action Planning (WRAP) and Illness Management and Recovery (IMR). WRAP has a specific focus on personal recovery and uses two trained experts by experience to guide group sessions (33). It may therefore miss the added value of a clinician and blended care. IMR focusses on supporting illness self-management strategies and uses individual or group sessions, homework assignments and the involvement of significant others (34). Afterwards, online facilities were added to create a blended version (35). IMR combines a focus on symptomatic recovery and personal recovery and takes 9 months to complete. It is therefore more difficult to use as a supplement to existing care. Furthermore it has not been developed as a blended program with guidance by both an expert by experience and a clinician. To the best of our knowledge, a program that is both designed to support personal recovery of patients with MDD and effectively combines support from family and peers, the involvement of experts by experience and the use of professional (blended) care is not yet available. To fill this gap, the program Storytelling and Training to Advance Individual Recovery Skills (STAIRS) was developed by a project group consisting of five clinicians from different disciplines and five experts by experience.

Because STAIRS is a newly developed program, a study into its feasibility is important before an effect study is carried out. This evaluation study aimed to examine the feasibility of the STAIRS program by assessing participants' and trainers' experiences with the program's acceptability and usability. Here, acceptability was defined as the level of appreciation participants and trainers had for the used content and didactics in STAIRS and the extent to which the program met the needs of the participants. Usability was defined as the extent to which participants and trainers felt that STAIRS is organized in a way that effectively and efficiently promotes the personal recovery process of participants.

## Materials and methods

### Study design

A qualitative evaluation study was used to investigate the acceptability and usability of the STAIRS program, based on the perspectives of patients and trainers. Experiences with the STAIRS program were collected from five participating patients with questionnaires and a focus-group interview, as well as from four trainers using semi-structured interviews. The conduct of the study and reporting of its results were guided by the COREQ standards for reporting qualitative research (36).

### Intervention

Characteristics of the STAIRS program are listed in Table 1. STAIRS focuses on the exchange of experiences between peers and on learning practical skills. The STAIRS program consists of 10 group meetings and the use of a private online environment. Each group meeting focuses on a specific topic (except for the first introductory meeting) and is guided by a clinician and an expert by experience. The following topics are addressed: (1) effects of depression and treatment; (2) structure; (3) (self) stigma; (4) self-image; (5) meaning of life; (6) connection to others; (7) physical health; (8) relaxation; and (9) preventing relapse. These topics were chosen, because they can each affect participants' daily functioning, social functioning and the way in which participants have come to see themselves since their depression. Each group meeting has a fixed structure. First, participants reflect on the past week, after which a new topic is introduced by the expert by experience. This is followed by two exercises, designed to address this topic. For example, in the meeting addressing structure participants fill out their current and their desired weekly schedule. Group discussions are encouraged by the trainers to foster the exchange of experiences between participants in addressing identified difficulties. Finally, meetings end with homework preparations. The homework assignments are intended to further practice what has been learned in the meetings. For example, participants are asked to have a conversation about their depression with a significant other using a specifically designed card game. To support participants in between meetings, a private online environment is available, containing an overview of the program, information on the different topics, the homework assignments and a forum in which they can share experiences and support each other in between meetings. The meetings were designed to be face to face. Due to the COVID-19 pandemic, the program meetings had to be conducted online, after the introduction meeting which was face to face.

**Table 1:** Characteristics of the STAIRS-program.

Topics of the meetings	Introduction/acquaintance meeting 1. Effects of depression and treatment 2. Structure 3. (self) Stigma 4. Self-image 5. Meaning of life 6. Connection to others 7. Physical health 8. Relaxation 9. Preventing relapse
Format of the meetings	Duration: 90 minutes Introduction of the theme Experience story referring to the theme Exercise Break (10 min) Second exercise Discussing homework assignments
Examples of exercises	Role-play A discussion in a subgroup Preparing for a conversation with a loved one Filling out one's current and desired weekly schedule
Examples of homework assignments	Ask feedback from significant others about one's qualities Try two activities aimed at relaxation Reflect on (experience with the) assignments using the online private community React online on two other peers in the group
Online content	Workbook Videos and texts used in the session Additional information (e.g. links to books, video's, podcasts)

### Setting and participants

The study was conducted between September 2020 and December 2020 at the University Center for Psychiatry (UCP); an academic mental healthcare service treating patients with, often severe, treatment-resistant disorders. Patients were recruited from the UCP as well as through the website “Alles goed” (www.allesgoed.org) that hosts personal stories of people who have (had) a depression and is targeted to people who struggle with the consequences of depression in their lives. Treating clinicians within the UCP were informed about the study and asked to refer eligible patients. On the website of “Alles goed”, information about the study was shared including contact information. Patients were eligible if they were (a) aged between 18 and 65 years, (b) in the last phase (recovery phase) of treatment for a diagnosed major depressive disorder, and (c) had no more than mild depression, as shown by a score of <25 points on the Inventory of Depressive Symptomatology—Self Rated (IDS-SR). Exclusion criteria were: (i) insufficient command of the Dutch language; (ii) having cognitive problems or indication of an IQ <80; (iii) not having a computer or smartphone; and (iv) awaiting referral to a different mental healthcare service for other mental problems (e.g., anxiety or developmental disorders).

In total, seven outpatients were referred, and two enlisted through the website. All eligible patients had an intake by one of the clinicians and one of the researchers to provide them with more in-depth information about the program and to check the in- and exclusion criteria. One patient was unable to use a computer or smartphone and therefore excluded. After the intake, patients decided whether they wanted to participate in STAIRS and provided informed consent. Of the eight participants who started the training, five completed the program. Two participants could not finish the program because of comorbid disorders requiring treatment. One participant needed surgery, which meant that participation in STAIRS could no longer be sustained.

Two STAIRS trainers were recruited from the project team that developed the STAIRS program. In addition two experts by experience were recruited as trainers. All four trainers were interviewed as part of the current study.

### Data collection

Acceptability and usability of the STAIRS program is examined with self-report questionnaires, semi-structured interviews and a focus group by asking participants and trainers about their experiences with the used *content, didactics* and *organization*. Measurements were done halfway through (T1) and at the end of the program (T2). In an iterative data-collection process data were alternately collected from participants and trainers. See Table 2 for an overview.

**Table 2:** Overview of measurements.

	**Every session**	**Halfway of program (T1)**	**End of program (T2)**
Participants		Self-report questionnaire	Self-report questionnaire Focus group interview
Trainers	Short questionnaire		Semi-structured interview

At T2, first the self-report questionnaires were administered, second the semi-structured interviews and third the focus group interview.

The researchers developed a self-report questionnaire for the participants, in which the experiences with the content, didactics and organization of STAIRS were questioned. This questionnaire contained 29 open questions, such as: “Which meeting was most valuable to you and why?” and “How do you asses the qualities of the trainers?” The questionnaires were administered at T1 and T2.

To collect data from the trainers, a short questionnaire was developed. All four trainers were asked to complete this questionnaire (containing 6 open questions) after each meeting, in which they were asked about their evaluation of the meeting, e.g., “What exercises did or did not work?” and “Did the exercises contribute to achieving the aims of the meeting?” The responses on these questionnaires were used to prepare the semi-structured interviews with the trainers at T2. An interview guide was developed for these interviews, containing questions like “To what extent were the working methods of this meeting workable?” and “To what extent did the different elements contribute to the intended aim and the structure of the meeting?” Furthermore, questions were asked about time investment, the trainer's manual and collaboration between trainers. Three interviews were conducted face to face at the UCP; one interview was conducted online using MS Teams. All interviews were conducted together by two interviewers; DW (MSc, male, lecturer at a university for applied sciences and PhD candidate) and JS (MSc, female, clinical psychologist at the UCP). Interview durations ranged from 50–140 min.

The last step in data collection was a focus group with the participants, organized and moderated by DW and JS in order to collect more in-depth information about the participants' experiences with the program. The five participants who completed the program participated in the focus group interview, which was conducted online using MS Teams and had a duration of 90 min. DW did not know the participants in person. JS had met the participants once during the intakes prior to the start of the program. A topic guide for the focus group interview was developed, based on data from the participants questionnaires and trainers' interviews. Topics included: themes and structure of the program; perceived effects; the usability of the online content and the online community; appreciation of social support during the program; opinion on quality of the trainers; working methods and exercises; duration/frequency; and suggestions for improvement of the program. After each topic a summary of what had been said was presented to the participants in order to check validity.

### Analyses

The Framework Approach (37) was used to analyse the data. First, a framework was built, based on data from the participants' questionnaires, including verbatim responses to the open questions and the researchers' summaries of each question. This framework was then used to adjust the semi-structured interviews and develop a topic guide for the focus group. The interviews and focus group were audio-recorded and then transcribed verbatim. The transcripts were independently open-coded by two researchers (DW and JS) using Atlas.ti (version 9.0.15). We started with three deductive codes (content, didactics and organization). Then, 24 inductive codes were found, which formed the basis for a second framework in which the qualitative data of the participants and trainers were summarized. The next step in the framework approach is “mapping and interpretation,” in which the data were further clustered, summarized and abstracted into four main- and six subthemes.

All steps in the process of qualitative research, including peer debriefing to ensure validity of the study data, were prepared and supervised by a senior qualitative researcher (MA; PhD, social scientist).

## Results

### Main results

**Figure 1:**
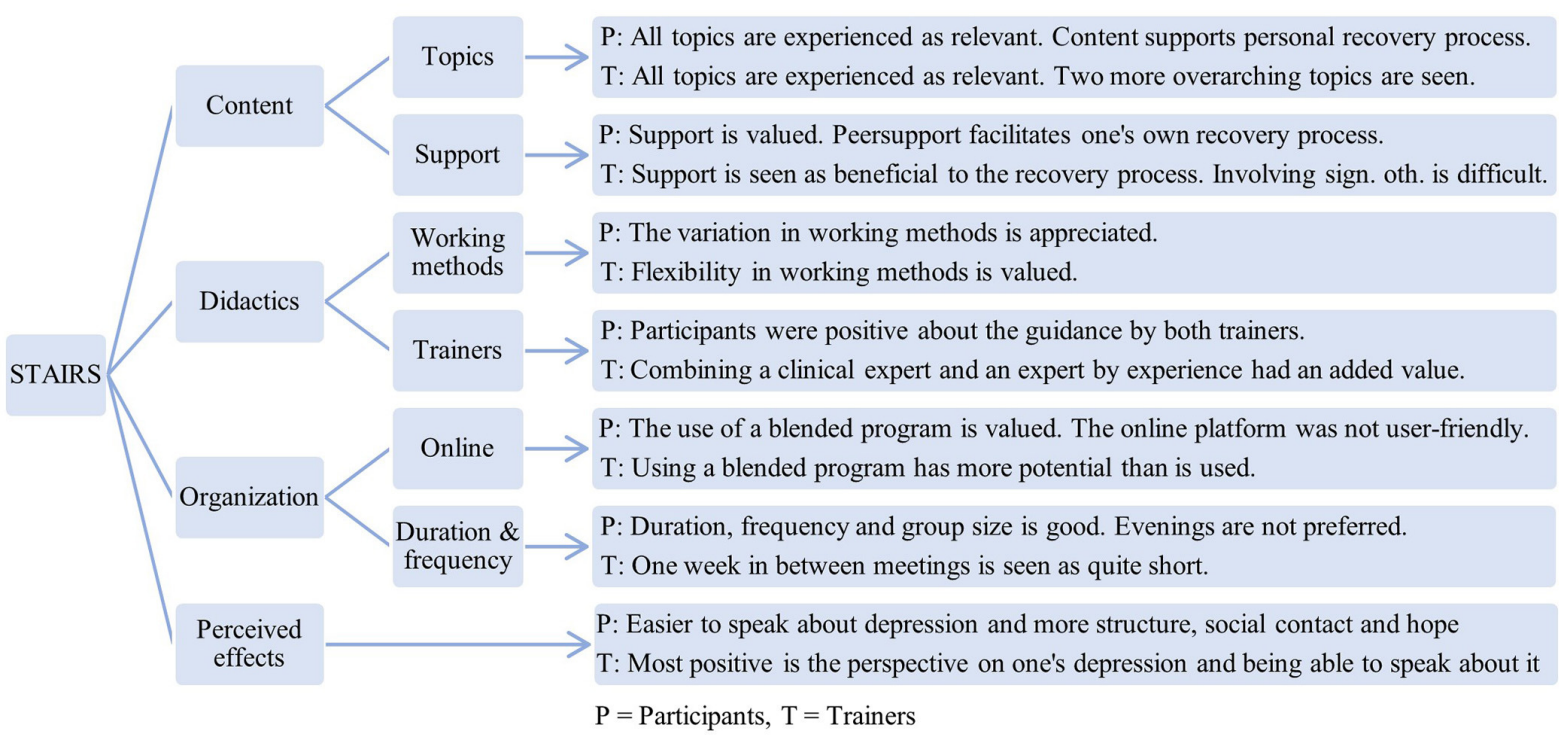
Combined results from all questionnaires, interviews and focus group interview.

Acceptability and usability of the STAIRS program were examined, based on participants' and trainers' experiences with three main themes: content; didactics; and organization of the program. Quotes (translated from Dutch to English by DW and JS) are used to illustrate the results. From the results in the framework, six subthemes were identified that could be categorized under these main themes. Furthermore, a fourth main theme was identified: perceived effects. Figure 1 shows an overview of the results.

### The content of the program

#### Topics

All topics addressed in STAIRS were seen as relevant for the personal recovery process by both the participants and trainers. What topic was most relevant to whom differed between participants, but all topics were seen as important to address the possible lasting consequences of a depression. For example, Participant 5 stated: “*I was already enthusiastic about the topics beforehand. These are areas that people with depression encounter*” and Trainer 2 stated: “*There were no topics that did not fit in the program. Some topics were not very relevant to some people, but each topic was relevant to someone*.” The topic of “structure” appealed the most to participants, because it helped them to find and maintain a healthy week schedule, which was perceived difficult by most participants. Trainers agreed on the importance of this topic and regarded structure as one of two overarching topics in the program. According to the trainers this topic could be expanded to include elements of the topics “physical health” and “relaxation.” The second overarching topic that trainers identified was “connection to others,” where a clear link was seen with the topics “(self) stigma,” “self-image” and “meaning to life”.

#### Support

Participants valued the support by peers. They experienced talking with peers as supportive and helpful to gain insight into their own situation. Participants indicated that the added value of peer contact lies in the ability to really share experiences, support each other and have more informal contact. For example, Participant 4 said: “*You understand each other, because you are, after all, fellow sufferers*.” Trainers saw mutual understanding between peers and believed this to be an important aspect of the program, because it helped to gain more insight into possible coping strategies, as is illustrated by Trainer 4: “*I think the most important aspect is recognition between participants. Someone tells how he/she dealt with a specific situation, and this can be a simple remedy, but others can then relate to the situation and learn from the ways others would deal with it*”.

The involvement of significant others was also valued, but turned out to be more challenging. Whether a significant other was involved depended on the availability of significant others in the participant's network and whether a participant chose to involve a significant other themselves. Trainers experienced difficulties with influencing the involvement of significant others, as they had no direct contact with them. Therefore, trainers suggested paying more attention to the involvement of significant others in the program. Trainer 2 said, for example: “*Perhaps we need to really put more emphasis on the significant others in the meetings. Because I think we involved them less than we had actually wanted*.” They mentioned that checking more often how a topic is related to significant others and actively involving significant others in the homework assignments could be helpful. Participants suggested inviting significant others to one of the meetings. Positive experiences were reported with a card game that facilitated a conversation between participants and significant others. This was mentioned by all participants as helpful in improving supportive contacts.

### The didactics of the program

#### Working methods

Overall, participants and trainers were positive about the variety in working methods and experienced these as suitable for the goals that had been set for each meeting. For example, Participant 8 said: “*There was a nice variation in having conversations through questions and more practically oriented exercises. I think it is the combination that made it good*.” Participants experienced the methods that were aimed at supporting the exchange of experiences to be effective, especially when the gained insights were then used in an exercise to practice specific skills. Furthermore, they valued the use of subgroups because this made it easier to become personal and to support each other. Participants were positive about the homework assignments, because these made them think about what was learned in the meeting and how they could use the learned skills/insights in their own situation. Some participants did mention that they sometimes struggled with planning their homework into their week's schedule. Trainers valued the possibility to be flexible with the different methods offered, because this gave them the freedom to adapt to what was going on during a meeting at a specific moment. For example, Trainer 1 said: “*Sometimes we skipped an exercise, because there was actually such a lively discussion among the participants. I think it is important to be able to respond to what happens in a meeting*”.

Participants received an online instruction manual and mentioned they were sufficiently able to work with it and found the information given in the manual helpful. However, trainers experienced the participants' and trainers' manual to be quite extensive and suggested shortening it and to clarify the link between each exercise and the aim of a meeting. For example, Trainer 1: “*Both the participants' and the trainers' manual have long texts in them, which I think is not all read by the participants. I think you should make it much shorter for the participants, with a short explanation for each meeting*”.

#### Trainers

Participants were positive about the trainers. They valued their approachable, enthusiastic, understanding, respectful and coaching attitude. For example, Participant 1 said: “*The guidance by the trainers was really good. They are easy to talk with and everything was clear*.” Participants found it important that trainers were able to recognize group processes and to guide these. This helped them to keep on topic and to feel safe and encouraged to exchange experiences.

The presence of an expert by experience next to the clinician was valued by the participants and trainers. Both play a different role in the program and complement each other. Trainer 1 formulated it as follows: “*I think the combination of professional and expert by experience is really very good. That definitely adds something both ways*.” Participants felt that the involvement of experts by experience had added value, because they had a better understanding of what it is like to struggle with the consequences of a mental illness. For example, Participant 4 said: “*I think an expert by experience can put his/her finger on something more often than someone who does not have this experience*.” Therefore, they found that the introduction of a topic was best done by the expert by experience. Furthermore, participants and trainers both found it important that experts by experience are able to look beyond their own experiences in order to use them as a facilitator in the recovery process of others. They felt the experts by experience had sometimes remained on the level of the participants too much, by also sharing own experiences without it contributing to the recovery process of the participants.

### Organization

#### Online platform

Participants valued the use of an online platform as an addition to the meetings, because this allowed them to keep in touch with each other between meetings. For some participants the ability to share experiences online by writing them down made it easier to share things that they found difficult to talk about. Participant 1 said, for example: “*I did notice, certain things that I wrote on the computer, that was really from my heart, and it would have been hard to speak about it for me. So, sometimes it is easier for me to write it down*.” Trainers indicated they felt the combination of an online platform and meetings has more potential than how it was used during this evaluation study. This was partly due to difficulties experienced using the website. Participants and trainers indicated that the online platform was not user-friendly enough because it was not accessible *via* a smartphone and several steps were needed before they could access the platform. Furthermore, it was sometimes unclear for them where to place a message. For example, Participant 5 said*:* “*I thought it was a lot of clicking through and clicking back to eventually end up somewhere. So I did not think it was a super clear site. I managed to put something on the right page, but that could be arranged a bit easier*” and Trainer 3 said*:* “*There are a number of people who have become quite skilled at the use of the online forum and were able to respond to each other and also give feedback in the next meeting, but there were also people for whom it remained a bit difficult*.” Suggestions were made by participants to make a simple mobile version with automated notifications when new messages are posted. Trainers suggested spending more time on explaining how to use the platform at the start of the program and to pay more attention in each meeting to what had been written online between the meetings.

Because of the restrictions caused by the worldwide COVID-19 pandemic, most meetings were online rather than face to face. Participants experienced some advantages of online meetings, but would have preferred the meetings to be face to face. They indicated that real contact was necessary to benefit from peer contact, which was perceived as more difficult online. Participant 4 said, for example: “*There were advantages of having the meetings online, but the assignments we did would have worked better face to face*.” Trainers also would have preferred face-to-face meetings, because this would have allowed for more active exercises and a better ability to gauge and influence group dynamics. Trainer 3 illustrated this as follows: “*The concerns are with the people who participate as spectators; how do you get them activated? This is really more difficult online*.” Furthermore, trainers mentioned that they experienced some time pressure to fulfill the different exercises in each meeting and felt this could partly be due to the fact that the meetings were conducted online.

#### Duration and frequency

Participants considered the duration and frequency of the meetings appropriate for the aims set per meeting. Weekly 2-h sessions allowed them to work well on a topic without it being too long and to maintain progress without having too little time to work on homework. Some participants mentioned they would have liked some kind of review halfway during the program. For example, Participant 8 said: “*You could also schedule a kind of extra session halfway in which you look back on what you have learned from the previous three sessions, for example. So you don't just do that at the end*”. Trainers indicated that a period of 1 week between meetings was quite short. In order to embed the homework assignments more into the meetings, they suggested starting a topic in the second half of a meeting and coming back to it in the first half of the next meeting. Both participants and trainers thought the evening was not the most suitable moment for the meetings, because energy levels were too low to make optimal use of the meetings. Participants preferred the meetings to be during the day and at the beginning of the week. Furthermore, both participants and trainers indicated that a group of eight participants is the right size, because this allowed for enough experiences to share but still feeling safe enough to be personal.

### Perceived effects

The main experienced effects that participants reported were: being able to speak more openly about depression; experiencing more structure in daily life; improvements in social contacts; and more hope for the future. For example, Participant 4 said: “*I have a better perspective on my depression and managed to get back the structure in my life. A difference is, for example, that people first came to visit me and now I make appointments and go to them*.” Participant 8 said*:* “*I have become aware again of what is important to me and I make more conscious choices in my life*.” Trainers saw similar improvements in the participants' lives: maintaining daily structure; more connection with others; and more openness about depression. Especially acknowledging difficulties caused by the depression and learning to speak about them was seen as an important effect, as is illustrated by this quote from Trainer 1: “*STAIRS supports [participants] in recognizing that there are things you run into and in learning to communicate about them*.” In addition, Trainer 3 said: “*A number of participants clearly stated they were very satisfied to have shared more with people in their environment… beyond the guilt and shame*”.

## Discussion

The aim of this study was to examine the feasibility of the STAIRS program by assessing its acceptability, usability and perceived effects from the perspective of participants as well as trainers. Important insights were gained from this study. First, regarding the acceptability of the intervention, both participants and trainers were positive about the used content and didactics in the STAIRS program. The topics addressed in STAIRS were perceived as relevant for the personal recovery process. The overall approach as well as the working methods chosen for the aim for each meeting were appreciated and seen as suitable. Participants valued the ability to share their experiences with peers and saw benefits in the combination of an expert by experience with a clinician. The involvement of significant others was also experienced as helpful, but turned out to be challenging. Second, with regard to the usability, the use of a blended program was valued. However, both participants and trainers suggested that the user-friendliness of the online platform should be increased in order to reach the full potential of a blended program. Furthermore, face-to-face meetings were clearly preferred over online as face-to-face contact was deemed important to truly benefit from peer contact and develop skills for personal recovery. Finally, regarding the perceived effects, both participants and trainers were positive about the effects of the STAIRS program. It appears to have had positive effects on the daily lives of participants and their contact with others.

It is interesting to note that participants experienced the progress they made in structuring their life as one of the most important outcomes of the STAIRS program. This is remarkable considering the fact that participants had finished most of their depression treatment prior to the STAIRS program, and most depression treatments also focus on improvement of structure (11). This could indicate that regaining control over one's week schedule after a depressive episode is a process that continues beyond symptomatic recovery. It is therefore important to address this further in personal recovery-orientated treatment for MDD. As reported in previous research, redefining daily life is a process, which can start only when the debilitating effects of a depression are over (38), so it could be that the timing of our intervention better suits this goal than when it is addressed earlier in treatment.

The findings of this study are consistent with previous research on personal recovery. The topics addressed in the STAIRS program, the use of a blended platform, peers and experts by experience, the involvement of significant others and the perceived effects, all relate to the processes described in the CHIME framework: i.e., connectedness, hope, redefined identity, meaning to life and empowerment (22). In particular, social support by significant others, peers and trainers was highly valued and experienced as helpful. This is in line with a recently published study among 158 patients with a serious mental illness, which also showed that involving relatives and significant others in treatment facilitated the personal recovery process (39). As reported in previous research, our study also demonstrates that involving significant others can be challenging, e.g., because no significant other was available or participants did not want to involve them (40–42). Suggestions made by participants and trainers to more closely involve significant others included: specifically designed homework assignments, more attention in meetings on how others are involved, and inviting them to join a meeting.

Regarding the choice to involve both a clinician and an expert by experience to act as trainers during meetings, STAIRS appears to be one of the first programs using this approach. Regular mental health care offers a range of evidence-based treatments for MDD, led by clinicians, focusing on symptomatic recovery. In addition, there is growing interest in the effects and valuation of support groups led by experts by experience to enhance personal recovery (26, 27). However, the combination of these perspectives on recovery in one program is rare (28, 38). This study shows that such a combination is valued by both participants and trainers, and may offer a relevant addition to support the recovery process of patients. By adding the program as a follow-up to regular treatment, it is possible to address the different dimensions of the recovery process of MDD patients (43). A prerequisite mentioned by both participants and trainers for involving experts by experience is that they are able to look beyond their own experiences and consciously use them as facilitators in the recovery process of patients, e.g., providing examples that match the different needs of different patients.

This study has several strengths. First, the intervention evaluated in this study combines different elements each of which are known to support personal recovery, but have not been applied jointly in a comprehensive program. Second, the evaluation was based on a systematic, iterative process, in which data were collected from both participants and trainers. This allowed for the use of different points of view during the systematic evaluation of the program and for the gathering of tips and suggestions from different respondents. Third, the validity of the findings was enhanced by using methods triangulation, combining information collected through questionnaires, interviews and a focus group. Fourth, analyses were done by multiple researchers and included peer debriefing, also enhancing validity.

This study also had some limitations. First, almost all sessions needed to be held online, because of restrictions due to the COVID-19 pandemic. Therefore, only experiences with online meetings could be evaluated, whereas STAIRS was originally developed to include face-to-face meetings. Although this highlighted the flexibility of the program and showed that online or a hybrid approach could still be viable depending on circumstances, the generalizability of the findings to a completely face to face version of STAIRS could be limited. Second, the sample size of this study was limited and not determined based on saturation. Results should therefore be interpreted with caution. Furthermore, a relatively small number of participants completed the final program evaluations. Interviews however showed that dropout was due to personal reasons and not related to effects of the program. Third, the researchers and trainers were involved in both the development and evaluation of the program. This could have led participants to give socially desirable answers, which in turn could have caused bias in the final evaluations. We tried to minimize this effect, by explicitly asking the participants for critical evaluations and suggestions for the future. Fourth, this study was done in a specialized academic mental health-care center, which could limit the generalizability of results to other populations.

In conclusion, the findings from this study indicate that STAIRS, a blended program using peer support and guidance by an expert by experience and a clinician, was feasible for patients recovering from MDD. Results indicate that the program can contribute to personal recovery and addresses a void in the field of depression treatment. Based on the very helpful suggestions made by participants and trainers, the STAIRS program and online platform will be further adapted and updated. After this, the efficacy of STAIRS will be investigated in an upcoming randomized controlled trial, comparing it to usual care with added psychoeducation.

## Data availability statement

The raw data supporting the conclusions of this article will be made available by the authors, without undue reservation.

## Ethics statement

The studies involving human participants were reviewed and approved by Medical Ethical Committee (METc) of the University Medical Center Groningen (no2020/031). The patients/participants provided their written informed consent to participate in this study.

## Author contributions

DW and JS: conceptualization, methodology, investigation, formal analysis, and writing of the original draft. EK and KW: conceptualization, critical review, and editing of the manuscript. MA: supervision of analyses, methodology, critical review, and editing of the manuscript. RS: conceptualization, critical review, editing of the manuscript, project administration, and supervision. All authors contributed to the article and approved the submitted version.

## Funding

This work was supported by Stichting tot Steun VCVGZ (project number: 255). DW obtained a personal grant from the Dutch Research Council (NWO; number 023.016.008) to obtain a PhD. The development of the STAIRS program has been supported by a grant from Agis Inovatiefonds (project number 2019-04).

## Conflict of interest

The authors declare that the research was conducted in the absence of any commercial or financial relationships that could be construed as a potential conflict of interest.

## Publisher's note

All claims expressed in this article are solely those of the authors and do not necessarily represent those of their affiliated organizations, or those of the publisher, the editors and the reviewers. Any product that may be evaluated in this article, or claim that may be made by its manufacturer, is not guaranteed or endorsed by the publisher.
